# Connection between Excessive Brain Worry and Bodily Disease

**Published:** 1877-11

**Authors:** J. J. Caldwell


### THE CONNECTION BETWEEN EXCESSIVE NERVE AND BRAIN WORRY AND BODILY DISEASE WITH PATHOLOGY AND TREATMENT OF CANCERS, TUMORS, ETC.

BY J. J. CALDWELL, M. D.

In a second article, on the development of carcinoma, published in Varchow's Archiv of June, 1872, by Professor Waldeyer, he substantially reaffirms the similarity of development between the so-called epithelial and other cancers, admitting that the elements of the small-celled brood are migrated white corpuscles, and that Kœster's view, that the cancer cylinders lie in the lymphatic passages, is very often correct, still holding firmly to the doctrine that "the cylinders themselves are always outgrowths from some normal epithelial structure with which they yet retain their connection.

We trust you will pardon us, Mr. President, when we modestly express our surprise that little or nothing, as far as our knowledge extends, occurs among the writings of the greater savans concerning the influence exercised by nerve currents or powers on cell formations, beneficially or detrimentally.

It occurs to us that the nerve force, in its effects and action upon cell structure, presents a close analogy to electro-plating. Indeed there is such an intimate alliance between these two mysterious forces as to render distinction between them inappreciable by the human intellect, except, as it was said by Sir Wilson Phillips, of London Academy of Science, that "the latter is incapable of restoring the vital spark to departed ones."

As an illustration of this analogy we would refer to the changes presented by the phenomena in electro-plating, with varieties of the battery, elements, and fluids. Contemplating these, it is not difficult for us to suppose that like irregularities could be effected through varying influences of nerve forces at the centres and peripheries. We candidly believe that until this hidden power, nerve influence, is more fully comprehended in its relation to cell formation, we will continue to remain in that condition, lately, in an able manner, defined by one of our popular journals, when referring to Prof. Brown Sequard's beautiful lecture, recently delivered on the "Mechanism of the Brain."

After referring to the doctor's undoubted ability and his demonstration of the seat in the brain of the volitional, sensorial and mental phenomena, the Journal remarks that we "are just as far from knowing anything about the mystery as was the devotee who gazed on the problem in the Temple of Sais, which told him, 'I am all that I was, all that is, and all that shall be.'"

Spite of the great research and application of modern science upon this subject, no man has yet been able to place his learned touch upon the precise spot of human cerebral anatomy, when in search for the seat of sensorial and mental phenomena, and exclaim *Eureka!* It is to be feared that all, yea the most ardent devotee of science and searcher after the hidden truths and grand mysteries of our organism, will only be led to repeat, "I am all that I was, all that is, and all that shall be—*and my veil hath no man raised.*"

It was brain worry and not brain work that caused the death of the sceptered hermit of the rock-bound St. Helena. Like Prometheus of old, he died not from a vulture destroying his vitals, but the humiliation and chargrin of his fallen position fretted his proud and lofty spirit, thus through its excessive irritation developing that most formidable disease termed carcinoma of the stomach. Yes, he died from carcinoma of the stomach, caused alone by the ever-chafing mental chains. While at liberty, his ceaseless and tireless mental energies only seemed to make him more and more patient. For this man, without a model and without a shadow, could never brook this continuous irritation, which so reflected itself as to produce that horrid pathological monstrosity called cancer. This is not an isolated case, for we may cite from many authors of our day, cases directly illustrating the effect of mental irritation in producing bodily disease. Among others we may quote from the able article written by Fernand Papillon, entitled, "The Pathology of the Passons," wherein he declares that physicians have in all ages recorded the passions among the predisposing, determining, or aggravating causes of the majority of diseases, especially the chronic diseases. The work of the passsions might be compared to the operations of a beleaguered city. They set about overmastering health and life circumspectly and slowly, but their advance is always sure, and of the most disastrous, in effect, when in excess. We may mention love, anger, melancholy, hate, etc. In exaggeration, these may become poisonous both to soul and body; for there is one form of melancholy which is plainly a variety of dementia, and often comes under the notice of the physician. It is characterised by an incurable sadness, an irresistible love of solitude, a belief in a host of imaginary evils ever haunting the patient. "My body is a burning fire!" wrote a melancholic subject to a medical man, "my nerves are glowing coals! My blood is boiling oil! Sleep is impossible!" Here we readily see the connection between excessive mental irritation and bodily disease; for such a patient would soon succumb, perchance from brain softening, cancer of the stomach, blood-degeneration, or other serious malady of this earthly tabernacle, this feeble tenement of the hour, unless some remedy be applied, a remedy indeed directly appealing to the brain and nerve centres, or in the words of the immortal bard, when the physician "can administer to a mind diseased, or pluck from the memory a rooted evil."

### TREATMENT.

In this connection we would cite the remarkable success which the learned Althaus of London has met with in the treatment of cancerous tumors by electrolysis. Of sixty-three cases treated by him up to 1868, by electrolysis, fifty-two were non-malignant and eleven of the malignant kind. The total result of the former was sixty-two per cent. cured, twenty-six improved, and twelve unknown. The doctor remarks that almost all the unsuccessful cases occur in the commencement of his electrolytic practice, when the method of procedure had not been so well developed as it is now.

"The facts that the peculiar lancinating pains of cancer generally disappeared soon after the commencement of the treatment, and that, even in rapidly growing cancers, the growth is often checked by it, seem to show that, by steadily working in the same direction, a remedial agent so powerful as that of electrolysis may yet be found to be instrumental in overcoming the local manifestations of this terrible disease."

Besides Althaus we might refer to the following eminent names of those who have successfully treated these tumors by electrolysis: Benedikt, Billroth, Paul Bruns of Germany; Lawson Tait, Fagge, and Callender of England.

Mr. Lawson Tait treated a case of encephaloid disease of the femur by electrolysis, complete relief from pain, for four or five days, following each application, except the first. The intensity of the pain had been so great that it had been found necessary to administer hypodermic injections of from 20 to 24 grains of morphia daily.

Bruns gives a valuable analysis of the reported cases of *naso-pharyingeal polypus*, nine in number, treated by electrolysis; seven resulted in complete cure.

Neftel claims to have destroyed a number of cancerous growths with better results than usual in the way of recurrence, by means of electrolysis. He believes that the cancer cells, being of low vitality, are killed by the current, even when they are not in a position to be directly decomposed.

Dr. Gilman Kimball of Lowell, in his report of casses of uterine fibroids, treated by electrolysis, says that "he generally found it most convenient to attack the disease through the abdominal walls. In some cases, however, when the fibroid growth had projected itself downward so as to involve a portion, or, as it sometimes does, the whole of the neck of the uterus, he made the attack through the vagina, thrusting one electrode into the tumor in that direction, and the other into the upper part, through the abdominal parietes. He witnessed no ill effect, local or constitutional, following this operation. For some hours after, the patient suffers considerable pain through the pelvic region, also some feverish excitement, as denoted by a quickened pulse, thirst and increased sensibility in the direction of the tumor. These symptoms, however, are not lasting, and in a day or two the patient declares herself entirely free from suffering of any kind."

At a late meeting of the British Medical Association, Dr. S. M. Bradley, in a paper in which he attempted to establish the essential oneness in origin of all morbid growths characterized by the abnormal development of epithelial elementry such as scirrus, epethelium epulis, and common warts, maintained that, as electricity, by coagulating the albumen of a part, establishes a barrier to the onward march of the cell elements, it should therefore be employed in all cases of infiltrating tumors, when it is decided to eradicate the growth.

Dr. J. Byrne, of St. Mary's hospital, Brooklyn, N. Y., reports seventy-three electric cautery operations in uterine surgery, his success being remarkable, most of the cases being malignant or non-malignant tumors. The operations were performed in the presence of Dr. Marion Sems, Emmett, J. C. Nott, and other eminent gentlemen.

We have also to record our own testimony in behalf of the treatment by electrolysis, from our own experience and operations for the removal of morbid growths, which, as we have already shown elsewhere, have been quite numerous. We will close this, we fear already too lengthy and tedious paper, by remarking that the study and practice of electro-therapeutics now claim the attention of the most eminent medical minds; of "men whose position and abilities inspire the confidence of their fellow-practitioners, and who are doing their utmost to rescue this specialty from the hands of unscientific men, to place it in the proper position. A great deal of good and careful work is now going on in this specialty, in all parts of the world, and the value of electricity, as a therapeutic agent, may be said to be slowly but steadily increasing." "If practitioners in general would make themselves more familiar with this therapeutic agent, and ascertain what it is capable of accomplishing, electricity would become much more popular with the profession than it has been."

